# Germline and somatic mutations of multi-gene panel in Chinese patients with epithelial ovarian cancer: a prospective cohort study

**DOI:** 10.1186/s13048-019-0560-y

**Published:** 2019-08-31

**Authors:** Wenhui Li, Di Shao, Lei Li, Ming Wu, Shuiqing Ma, Xianjie Tan, Sen Zhong, Fengming Guo, Zhe Wang, Mingzhi Ye

**Affiliations:** 1 Department of Obstetrics and Gynecology, Peking Union Medical College Hospital, Shuaifuyuan No. 1, Dongcheng District, Beijing, 100730 China; 2 BGI Genomics, BGI-Shenzhen, Shenzhen, 518083 China; 3 BGI-Guangzhou Medical Laboratory, BGI-Shenzhen, Guangzhou, 510006 China

**Keywords:** Epithelial ovarian cancer, Next-generation sequencing, Germline mutation, Somatic mutation, Homologous recombination deficiency

## Abstract

**Background:**

Multiple targeted gene sequencing is seldom performed in both germline and somatic testing for ovarian cancer. This study is to evaluate the specific genetic alterations, including both somatic and germline mutations, in Chinese patients with epithelial ovarian cancer (EOC) in a prospective cohort study.

**Materials and methods:**

Mutations in a customed 21-gene panel that included *BRCA1*, *BRCA2*, and 19 other tumor suppressor genes related to homologous recombination (HR) deficiency or non-HR deficiency were detected by targeted exon capture and next-generation sequencing (NGS) technology across all coding exons and exon-intron (±20 base pairs) boundaries. Patients were enrolled consecutively and unselectively without age or family history consideration. Sixty-two unselected patients with epithelial ovarian cancer were enrolled in our study to be tested for paired somatic and germline mutations. All patients were tested using a 21-gene panel that included *BRCA1*, *BRCA2*, *CHEK2*, *PALB2*, *BRIP1*, *TP53*, *PTEN*, *STK11*, *CDH1*, *ATM*, *BARD1*, *MLH1*, *MRE11A*, *MSH2*, *MSH6*, *MUTYH*, *NBN*, *PMS1*, *PMS2*, *RAD50*, and *RAD51C*.

**Results:**

Mutation analysis revealed that 77.4% (48/62) of patients carried one or more of 64 identified genetic alterations, including 19 germline and 45 somatic deleterious mutations. Twelve individuals shared both germline and somatic mutations. *BRCA* mutants existed in 17 of 62 (27.4%) patients. Of the 64 mutations detected, 46 (74.2%) were in 7 other HR or non-HR genes, including *TP53*, *PTEN*, *ATM*, *CHEK2*, *PALB2*, *RAD51C*, and *STK11*. In somatic mutation analysis, *TP53* showed frequent pathogenic or likely pathogenic mutations in 56.5% (35/62) of enrolled cases, among which six cases harbored a loss of heterozygosity.

**Conclusions:**

This is the first report of multi-gene panel testing for germline and somatic mutations among Chinese EOC patients, which revealed a broader deleterious variants than only *BRCA* testing.

**Registration:**

Registration No. NCT03015376, *clinicaltrials.gov*, registered on January 10, 2017.

**Electronic supplementary material:**

The online version of this article (10.1186/s13048-019-0560-y) contains supplementary material, which is available to authorized users.

## Background

Ovarian cancer is the third most common gynecological malignancy and the leading cause of mortality from cancer among females [1]. According to data from the Surveillance, Epidemiology, and End Results (SEER) program, ovarian cancer represents 1.3% of all new cancer cases, and there will be approximately 22,240 new diagnoses and 14,070 ovarian cancer-related deaths in the United States in 2018 [2]. The prevalence of ovarian cancer in China has increased in the past 10 years as well, with 52,100 new cases and 22,500 cancer-related deaths in 2015 [3]. Ovarian cancer encompasses a heterogeneous group of malignancies that vary in etiology and molecular biology. More than 90 % of ovarian cancers are epithelial, with the most common being serous carcinoma. The majority of patients are diagnosed at advanced stages, with 51% at stage III and 29% at stage IV, resulting in poor prognosis. The high mortality and resistance to therapy related to epithelial ovarian cancer (EOC) have promoted extensive study on its pathogenesis.

In general, tumorigenesis is a process driving a normal cell toward a malignant state resulting from multiple genetic alterations that comprise both somatic (acquired) and germline (inherited) mutations [4, 5]. As reported, hereditary backgrounds are shown in approximately 5 to 10% of invasive ovarian cancers [6–9]. In addition, inherited ovarian cancer may present as one of the autosomal dominant familial syndromes, i.e. hereditary breast and ovarian cancer (HBOC), site-specific ovarian cancer, and Lynch syndrome (hereditary nonpolyposis colorectal cancer) [10, 11]. International collaborative studies identified 15–20% of all EOCs to be associated with germline mutations [8, 9, 12, 13]. Moreover, most cases of inherited susceptibility to EOC are primarily related to germline mutations of *BRCA1* and *BRCA2,* which account for 95% of hereditary ovarian cancers and have been implicated in several cellular processes, including homologous recombination (HR), chromatin remodeling, regulation of the cell cycle, transcription, cell growth and differentiation. Carriers of such germline mutations obtain a lifetime risk of 55 to 85% for breast cancer and 15 to 60% for ovarian cancer [14–16]. However, only 20% of hereditary ovarian cancers have shown mutations in the locus of *BRCA1/2*. While clinical relevance in germline mutations of the *BRCA* gene is largely established, the full significance of somatic *BRCA* mutations is still unfolding. Whether harboring somatic *BRCA1* or *BRCA2* mutations has the same biological effects as their germline counterparts remains unclear.

In recent years, the advent of next-generation sequencing (NGS) technology has greatly promoted research in cancer genomics and cancer genetics [17, 18]. In addition, more genes have demonstrated close relevance in facilitating the onset of ovarian cancer. A better substratification of tumors based on their genomic profiles will contribute to the use of more targeted therapies. Thus, increased efforts have been made to research the molecular mechanisms underlying EOC. However, far less is still known about the genetic alterations of EOC in China. Although several small-size studies have previously reported the assessment of mutation profiles of susceptibility genes, there are no precise data about somatic *BRCA1/2* mutations and other related known breast or ovarian cancer suppressor genes among the Chinese population with ovarian cancers. We performed this research by enrolling unselected consecutive patients with EOC from the study center to screen for a 21-gene panel using the targeted capture and NGS approach.

## Materials and methods

### Study subjects

This is an interim analysis of the study “Inherited Susceptible Genes Among Epithelial Ovarian Cancer” (registration No. NCT03015376, *clinicaltrials.gov*). From March 1, 2017 to December 1, 2017, a cohort of patients with EOC (including EOC, carcinoma of fallopian tube and primary peritoneal carcinoma) who received oncogenetic counseling at the gynecologic clinic and surgical treatment at the gynecological ward in our center were consecutively included in our study. They were enrolled prospectively and unselectively without considering their ages, familial cancer histories, histological subtypes, or therapeutic processes. Basic information on patients regarding individual age, neoplasm staging and grade, and histopathological type was retrieved from medical records. Exclusion criteria included non-EOC histology and without paired tumor tissues. This study was approved by the Institutional Review Board (IRB) of study center (Registration No. ZS-1245). The Chinese Human Genetic Resources Management Office of the National Ministry of Science and Technology approved this study (registration No. [2017]1901, http://www.most.gov.cn/bszn/new/rlyc/jgcx/index.htm). All patients gave informed consent before enrollment.

All mutations were analyzed using blood samples and ovarian carcinoma tissue, which were collected before any surgical or chemotherapy treatment. Patients with neoadjuvant chemotherapy were excluded from the analysis.

### Next-generation sequencing

Genomic DNA (gDNA) was extracted from participants’ peripheral blood samples and from paired frozen tissues or formalin-fixed paraffin embedded sections using the Qiagen DNeasy Blood & Tissue Kit (Qiagen, Hilden, Germany) per protocol. Indeed, all the issues for analysis in this study were all from paired frozen ones. DNA concentration and quality were assessed by Qubit (Life Technologies, Gaithersburg, MD, USA) and agarose gel electrophoresis. gDNA (250 ng) was used for sequencing library construction as previously described [19]. Briefly, the gDNA was fragmented randomly by Covaris to generate gDNA fragments with a maximum of 250 bp and then subjected to three enzymatic steps: end-repair, A-tailing and BGISEQ-500 sequencer (BGI Genomics, Shenzhen, China) adapter ligation. DNA libraries were purified with Agencourt Ampure XP beads (Beckman-Coulter, Indiana, USA), and PCR was performed, during which a unique 8 bp barcode was added to label each sample. Five to ten PCR products were pooled equally and hybridized to a custom hereditary cancer panel. The hybridization product was subsequently purified, amplified and qualified. Finally, sequencing was performed with a paired-end 50 bp and 8 bp barcode on a BGISEQ-500 sequencer following the manufacturer’s protocols.

### Sequencing data analysis and mutation calling

Raw data generated by the BGISEQ-500 sequencer were first filtered by SOAPnuke to exclude reads with low quality. The clean reads were then aligned to the reference human genome (UCSC hg19) using the BWA MEM algorithm. Single-nucleotide variants (SNVs) were detected by Genome Analysis Toolkit (GATK) Unified Genotyper. Small insertions and deletions (indels) were called using GATK Haplotype. Copy number variants (CNVs) were called using read-depth analysis. For tumor tissue samples, somatic SNVs and indels were identified by GATK Mutect 2, and CNV status was calculated by Contra. All above variants were further filtered by quality depth, strand bias, mapping quality and read position. Each variant was finally annotated with respect to gene location and predicted function in Human Genome Variation Society (HGVS) nomenclature and was ready for interpretation.

### Data interpretation

Interpretation focused on the variants in 21 selected ovarian cancer susceptibility genes (*BRCA1, BRCA2, CHEK2, PALB2, BRIP1, TP53, PTEN, STK11, CDH1, ATM, BARD1, MLH1, MRE11A, MSH2, MSH6, MUTYH, NBN, PMS1, PMS2, RAD50, RAD51C*). These 21 genes were selected through the National Comprehensive Cancer Network (NCCN) guidelines and published research articles, which include core genes in the Fanconi anemia (FA) pathway and HR genes. Variants were classified into the following 5 categories according to the American College of Medical Genetics (ACMG) recommendations: class 1, benign; class 2, likely benign; class 3, variant of unknown significance (VUS); class 4, likely pathogenic; and class 5, pathogenic [20]. Individuals with likely pathogenic or pathogenic variants were defined as having “pathogenic” variants. Every likely pathogenic and pathogenic variant was validated by specific qPCR and Sanger sequencing.

### Statistical analysis

In cases with paired peripheral blood and tumor tissue, mutations would be involved in the germline mutation group when both a germline and a somatic HR mutation were tested simultaneously. Categorical variables were described with respective frequency values, while continuous variables were described with the median and range values. All P values less than 0.05 by two-tailed mode were considered statistically significant. Statistical Product and Service Solutions (SPSS) Statistics 20.0 (IBM Corporation, Armonk, NY, USA) was used for all statistical analyses.

## Results

### Patient characteristics

From March 1, 2017 to December 1, 2017, a total of 62 individuals were recruited in our study: 48 individuals with primary carcinomas and 14 with recurrent carcinomas. The characteristics of all patients are shown in Table 1. The median age of diagnosis was 56 years (range 34 to 82); two patients (3.2%) were under 40 years of age. The majority of cases were at advanced stages (45/62 were at stage III/IV, 72.6%) and of serous histology (49/62, 79.0%). Several other types of ovarian cancers involved referred to endometrioid, clear cell, squamous cell, or mixed carcinoma, unclassified adenocarcinoma, malignant Brenner carcinoma, and mucinous carcinoma. Five patients had a history of breast cancer, and 27 cases had a family history of malignancy with at least one first- or second-degree relative who suffered from various types of cancers. Targeted capture and genomic high-throughput sequencing after library construction yielded a median 99.82% coverage; the percentage of targeted bases at > 30 depth was 99.32%.

**Table 1:** Demographic and clinical characteristics of study population (n = 62)

Clinical characteristics	N/n of Patients (%)
Age at diagnosis	
< 40	2/62(3.2%)
40~50	18/62 (29.0%)
50~60	20/62 (32.3%)
60~70	18/62 (29.0%)
≥ 70	4/62 (6.5%)
FIGO stage	
I	6/62(9.7%)
II	5/62 (8.1%)
III	36/62 (58.1%)
IV	9/62 (14.5%)
NA^a^	6/62 (9.7%)
Primary or Recurrent	
Primary	48/62 (77.4%)
Recurrent	14/62 (22.6%)
Tumor histopathology	
High-grade^b^ serous	48/62 (77.4%)
Clear cell	5/62 (8.1%)
Endometrioid	3/62 (4.8%)
Low-grade^c^ serous	1/62 (1.6%)
Mucinous	1/62 (1.6%)
Others^d^	4/62 (6.5%)
Self, in addition to ovarian cancer	
Breast cancer	5/62(8.1%)
No breast cancer	18/62 (91.9%)
Family history	
Breast cancer	3/62(4.8%)
Ovarian cancer	7/62 (11.3%)
Pancreatic cancer	1/62(1.6%)
Pancreatic cancer	1/62(1.6%)
Colon cancer	2/62(3.2%)
Other cancer	18/62(29.0%)
Breast or ovarian cancer	10/62(16.1%)

a: NA = Not Available

b: Grades 2–3

c: Grade 1

d: Other = one malignant Brenner, one squamous cell carcinoma, one mixed carcinomas, and one unclassified adenocarcinoma

### Overall mutation rate

**Fig. 1:**
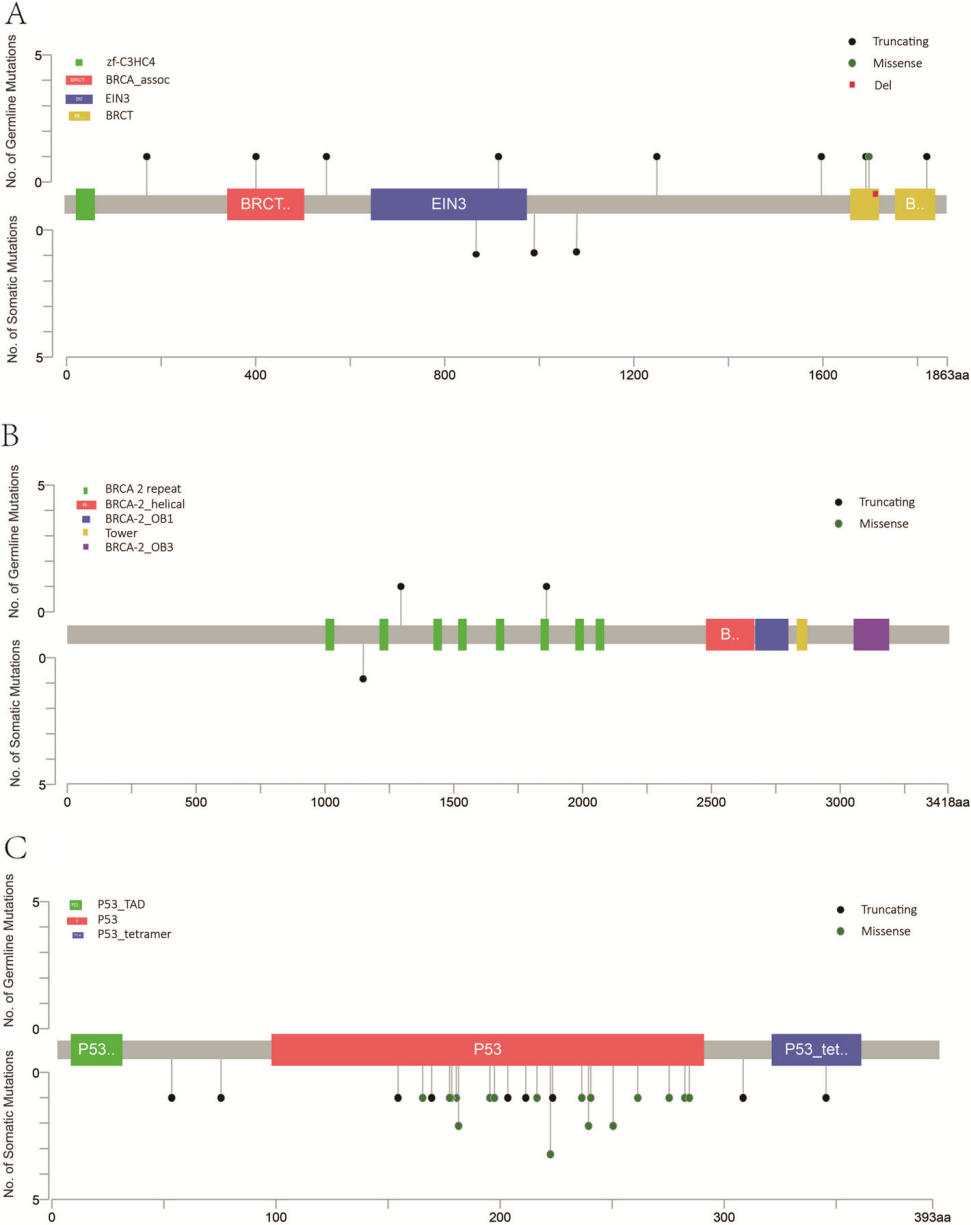
Germline and somatic pathogenic mutations loci in BRCA1 (**a**), BRCA2 (**b**) and PTEN (**c**) detected in 62 paired blood and tumor tissue samples

**Fig. 2:**
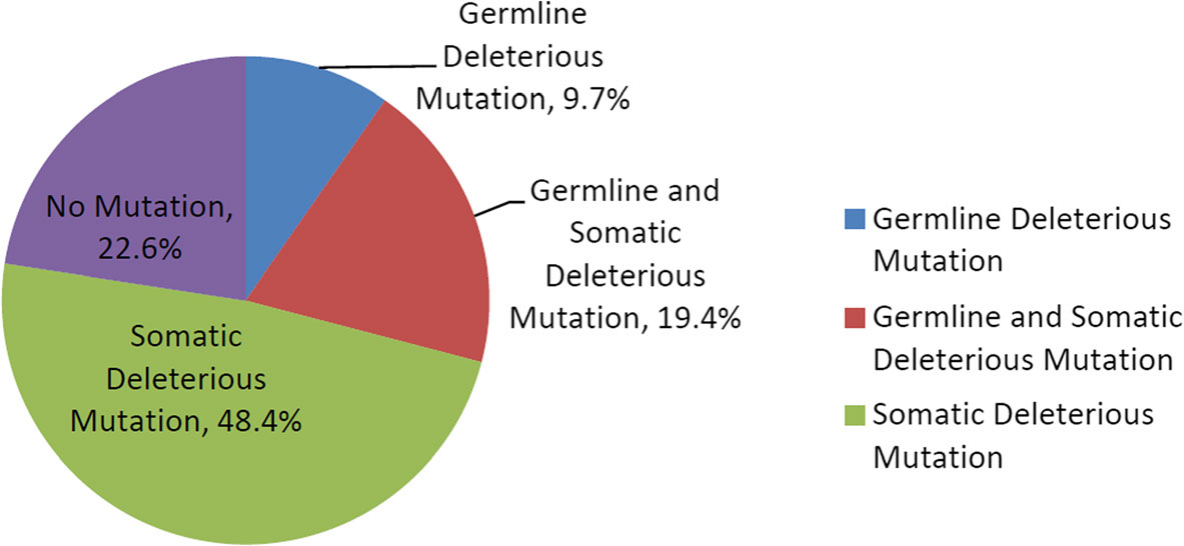
Overall germline and somatic mutations in 62 cases

**Fig. 3:**
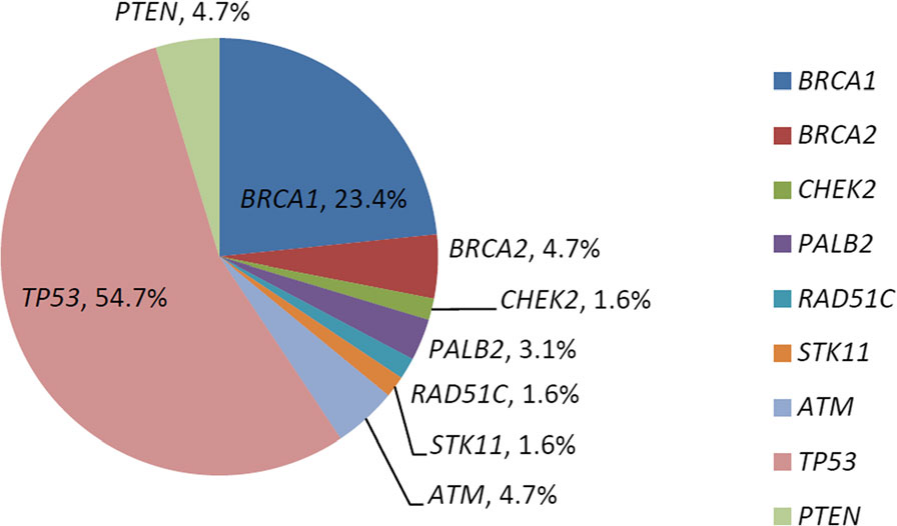
Deleterious mutations in germline and somatic mutations

Eighteen of 62 patients (29.0%) had a deleterious germline mutation in the 21-gene panel, and 42 (67.7%) had a somatic mutation (Additional file 1: Table S1). All the issues for analysis in this study were all from paired frozen ones before any surgical intervention or chemotherapy. Twelve individuals shared both germline and somatic mutations, and all of them were serous ovarian cancers, with 75% at advanced stages. One patient had more than one germline mutations (Additional file 1: Table S1, a HGSC patient of FIGO stage IIC, with deleterious *BRCA1* and *BRCA2* mutations). In summary, the proportion of cases with at least one deleterious mutation, whether it was germline or somatic, among all enrolled patients was 77.4% (48 of 62; Fig. 1 and Fig. 2). There were 64 germline and somatic deleterious mutations recorded, 15 of which (23.4%) occurred in *BRCA1*, 3 (4.7%) of which occurred in *BRCA2* and 46 (74.2%) of which occurred in 7 other HR or non-HR genes, including *TP53*, *PTEN*, *ATM*, *CHEK2*, *PALB2*, *RAD51C*, and *STK11* (Fig. 3). In cases with more than one mutation, two patients with high-grade serous ovarian cancers were recorded to carry 3 types of mutations from the 21-gene panel simultaneously.

### Germline mutations

**Fig. 4:**
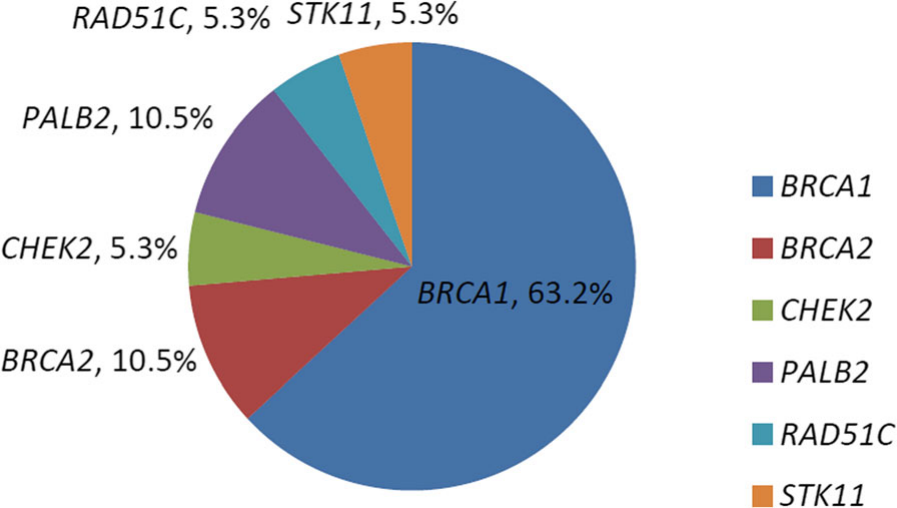
Germline mutations in 62 patients

Of the 62 total participants, eighteen subjects were identified as having 19 deleterious germline mutations in 6 different genes: *BRCA1*, *BRCA2*, *CHEK2*, *PALB2*, *RAD51C*, and *STK11*. The 19 deleterious germline mutations in these 6 tumor suppressor genes included 12 (63.2%) in *BRCA1*, 2 (10.5%) in *BRCA2*, and 5 (26.3%) in other genes that when mutated are potentially associated with an increased risk of ovarian cancer: 2 (10.5%) in *PALB2*, 1 (5.3%) in *CHEK2*, 1 (5.3%) in *RAD51C*, and 1 (5.3%) in *STK11* (Fig. 1 and Fig. 4 and Table 1). Interestingly, all 19 identified patients had high-grade serous ovarian cancers, and approximately half (8/18) had at least one ovarian cancer relative. Other types of ovarian cancer, by contrast, had not yet been correlated with any germline mutations. Of all the mutations detected, one individual, whose sister was also diagnosed with ovarian cancer, had a germline mutation in both *BRCA1* and *BRCA2*.

### Somatic mutations

**Fig. 5:**
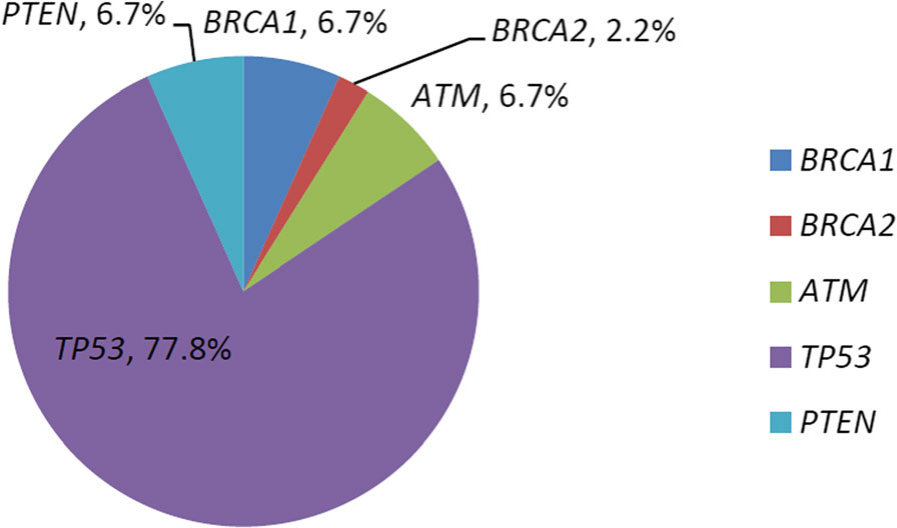
Somatic mutations in 62 patients

Abundant somatic mutations were detected in our 62 patients. A total of 42 (67.7%) cases were identified, with a total of 45 pathogenic or likely pathogenic mutations. All mutations were confirmed at the loci of 5 genes, including *BRCA1*, *BRCA2*, *TP53*, *PTEN*, and *ATM* (Fig. 1 and Fig. 5). *TP53* showed the highest frequency at 35/45 (77.8%) and was most likely to have somatic mutations among these enrolled patients with EOC, but no germline *TP53* mutation was found. Six cases with *TP53* mutations had loss of heterozygosity (Additional file 2: Table S2). All these cases were of serous type including one of mixed type. *TP53* mutations were not limited to high-grade serous carcinomas; they were also observed in other subtypes, including 1/3 endometrioid carcinoma, 1/1 malignant Brenner carcinoma, and 1/1 low-grade serous carcinoma. Another 10 mutations occurred in another 4 genetic sites: 3 (6.7%) in *BRCA1*, 3 (6.7%) in *PTEN*, 3 (6.7%) in *ATM*, 1 (2.2%) in *BRCA2* (Fig. 5). Therefore, the risk of somatic mutations in the *BRCA* gene among all cases was 6.5% (4/62). Details of deleterious mutations are summarized in Additional file 2: Table S2. Classes of damaging events included missense mutations with demonstrated effects on protein function, nonsense mutations, and other protein-truncating mutations.

### Mutations in nonserous histology

Fourteen cases (22.6%) had tumors of nonserous histology, including 5 clear cell, 3 endometrioid, and 1 squamous cell carcinoma, 1 mixed serous with carcinosarcoma, 1 mucinous carcinoma, 1 malignant Brenner carcinoma, 1 unclassified poorly differentiated adenocarcinoma and 1 unknown histology. Seven of 14 (50%) nonserous cases had a deleterious somatic mutation (Table 2), while no germline mutation was detected in the 21-gene panel. Correspondingly, 41/48 (85.4%) serous cases had either a germline or somatic deleterious mutation (*P* = 0.0047). Moreover, in patients with nonserous ovarian tumors, no *BRCA1/2* mutations were identified in our study, and existing somatic mutations focused on *PTEN*, *TP53* and *ATM* in 2 clear cell, 2 endometrioid, and 1 mucinous carcinoma and 1 malignant Brenner carcinoma.

**Table 2:** Nonserous cases with somatic mutations

Histology	Stage	Somatic mutation(s)
Clear cell	IIIC	*PTEN,* c.797_c.801 + 4delAAAAGGTTT
Clear cell	IC	*ATM*, c.8343_8344delTA
Endometrioid	IC	*PTEN,* c.302_305delinsACC/c.406delT/c.635-1G > T
Endometrioid	IIIB	*TP53,* c.451delC
Mucinous	NA	*ATM*, c.748C > T
Malignant Brenner	IC	*TP53*, c.527G > T
Mixed	NA	*TP53*, c.532C > G

### Mutations and family history

According to the available medical information, 6 of all recruited patients in this study suffered from other malignancies besides ovarian cancer: 5 had breast cancer and 1 had thyroid cancer. In addition, 27 patients had at least one first- or second-degree relative diagnosed with malignant tumors. Ten individuals (16.1%) with relatives developing breast and/or ovarian cancer were classified as having HBOC-related family history. A family history of esophageal, gastrointestinal, urinary and lung cancers was also confirmed in 17 cases. Considering the 14 patients with familial and personal histories of HBOC-related tumors, 13 high-grade serous and 1 high-grade endometrioid carcinomas were covered, and 92.9% (13/14) had either somatic or germline mutations (Additional file 3: Table S3). The ratios of *BRCA1* and *BRCA2* mutations detected were 9/14 (64.3%) and 1/14 (7.1%), respectively, which was much higher than the average ratios of *BRCA1* (15/62) and *BRCA2* (3/62) mutations in all enrolled cases. In addition, *TP53* showed frequent somatic mutations in these specific patients with a ratio of 9/14 (64.3%), but a positive association could not be proved with familial aggregations of breast or/and ovarian cancer suggestive of HBOC syndrome.

### Mutations and resistance to platinum

According to the effectiveness of platinum, there were 9 patients primarily resistant to the platinum (with recurrence within 6 months after the platinum-based chemotherapy). Germline *BRCA1/2* mutations carriers had fewer patients of resistance to platinum than non- *BRCA1/2* mutations carriers but without statistical significance (1/13 [7.7] vs 8/49 [16.3%], P = 0.390). Besides, germline deleterious mutations carriers had less patients of resistance to platinum than non-deleterious mutations carriers but without statistical significance (2/18 [11.1%] vs 7/44 [15.9%], P = 0.481).

## Discussion

Recent advances in the study of the potential molecular mechanism of ovarian cancer contribute to distinguishing familial cancer susceptibility and susceptible populations; moreover, they are meaningful and helpful for discovering effective predictive biomarkers and possible useful therapeutic procedures and drugs, such as poly ADP-ribose polymerase inhibitors (PARPi) and anti-angiogenic agents. For deleterious *BRCA1/2* or other HBOC-related mutation carriers, genetic counseling and prophylactic risk-reduction treatment under certain indications will be greatly beneficial in decreasing the incidence of ovarian and breast cancer. For patients, genetic testing for potential pathogenic sites can provide insight for initial or post-relapse treatments for better prognosis. Therefore, genetic testing has become an integral and important part of clinical practice, although it is more generally limited to *BRCA* genes and to women with specific family histories. In view of the small number of studies and clinical trials, the gene mutation spectrum associated with ovarian cancer and the corresponding mutation characteristics in the Chinese population and patients have not been fully understood and explained. Robust concordance data for germline and somatic mutation testing are currently lacking as well. In our study, we used a 21-gene panel to detect mutations including in *BRCA1/2* and other tumor suppressor genes that potentially predispose an individual to breast or ovarian cancer to improve knowledge regarding the relationship of a patient’s gene mutation spectrum to ovarian cancer risk in China.

According to statistics from the Cancer Genome Atlas (TCGA), homologous recombination defects (HRDs) were reported in approximately 50% of high-grade serous ovarian carcinomas [21]. There are also patients deficient in HR due to epigenetic inactivation of *BRCA1/2* or *BRCA1/2*-independent defects in the HR pathway. The prevalence of *BRCA* mutation observed in our study population was 27.4% (17/62), which was relatively high compared with the previously reported rates of 5 to 29% and was similar to the percentage in China of 28.5% obtained from a nationwide multicenter prevalence study on *BRCA* mutation [4, 12, 22–39]. It is believed that unselected *BRCA* testing in high-grade EOC is cost-effective and can identify 50% more carriers than can earlier family history or clinical criteria-based testing. As data from studies reported, of women with inherited mutations, 9–30% had no family history of breast or ovarian cancer. The proportion was considered to be underestimated due to limited genetic testing in patients without a strong family history. In our study, the proportion was 55.6% (10/18), which seemed much higher. Therefore, academic societies, such as the Society of Gynecologic Oncology (SGO) [40] and the NCCN, [41] have recommended that germline *BRCA1/2* testing should be performed in all patients diagnosed with EOC regardless of their family history.

*TP53,* as a key tumor suppressor gene, has been reported to frequently mutate in many cancers, and the frequency varied considerably between cancer types, which was below 10% in hematopoietic malignancies but up to 70% in head and neck cancers [42]. In serous ovarian cancers, *TP53* somatic mutation was rather common and was detected in approximately 40–60% of advanced ovarian cancers [43]. Consistent with data from previous reports, we identified the frequency of a somatic mutation at *TP53* to be 56.5% (35/62) among all enrolled cases. In addition, more evidence has emerged regarding variations in HR genes other than BRCA1/2 or non-HR tumor suppressor genes, including germline or somatic mutations in *ATM*, *PALB2*, *RAD51C*, *RAD51D*, *BARD1*, *BRIP1*, *CHEK1*, *CHEK2*, *MRE11A*, and *PTEN* [8, 12, 44–46]. Evidence was provided for the newer ovarian cancer-associated genes, such as *RAD51C/RAD51D/BRIP1,* which were associated with ovarian cancer risks of 5.8–11% and already met the requirement for risk-reducing salpingo-oophorectomy, which is recommended for patients with > 5% ovarian cancer risk [47]. Therefore, further studies to complete the molecular image of ovarian tumorigenesis and development would contribute to individualized and comprehensive therapy and lead to a better understanding of tumor heterogeneity.

Patients with *BRCA1/2*-associated ovarian carcinomas presented better survival results than noncarriers [13, 48–52]. Based on a summary and analysis of 26 observational studies that included 3879 invasive epithelial ovarian cancers, the 5-year survival rates for *BRCA1* mutation carriers, *BRCA2* mutation carriers and noncarriers were reported as 44, 52 and 36%, respectively [51]. The survival advantage of patients with positive *BRCA1* (HR, 0.78; 95% CI, 0.68–0.89; P < 0.001) or *BRCA2* (HR, 0.61; 95% CI, 0.50–0.76; P < 0.001) mutations was obvious. As an interim analysis of the study, we couldn’t achieve the survival outcomes in this cohort. However, current data had illustrated patients with germline *BRCA* or deleterious mutations may have less cases of resistance to platinum, which was the most principal regimens for EOC. Due to the limited case number, we couldn’t judge whether this discrepancy would impact the survival differences.

In our study, we practically used paired frozen tissues rather than formalin fixed paraffin embedded (FFPE) sections for the analysis of somatic variants. However, Currently, no evidence could show justify that results of sequencing analysis between these two different fixation/storage methods (frozen vs formalin fixed) are comparable. Duration and method of FFPE tissues may also impact quality of genomic DNA and gene expression changes evaluated by NGS. This is the main consideration we didn’t utilized the FFPE tissues.

The most limitation of current report is the limitation of case number. An eventual analysis of our study would provide more sufficient data of Chinese patients. Lack of analysis on *RAD51D*, *EPCAM* and other essential hereditary ovarian cancer genes is another limitation of our study. An feasible expanded panel of more relevant HR genes is needed to generalize in EOC population. However, such application is lack of the cost-effectiveness analysis.

## Conclusion

In conclusion, 77.4% of patients with EOC were identified with at least one somatic or germline mutation. Deleterious germline and somatic mutations were screened out in 29.0 and 67.7% of patients, respectively. *BRCA* showed the most frequent mutation in germline testing, while *TP53* showed the most frequent mutation in somatic testing. Multi-gene panel revealed more deleterious variants than only *BRCA* testing.

## Additional files

Additional file 1: **Table S1.** Cases with deleterious germline and somatic mutations. (DOCX 22 kb)

Additional file 2: **Table S2.** Cases with deleterious mutations. (DOCX 28 kb)

Additional file 3: **Table S3.** Cases with familial and personal histories of HBOC-related tumors. (DOCX 16 kb)

## Data Availability

All data of the study has been contained in the supplemental tables.
