# Supplementary material for: Germline and somatic mutations of multi-gene panel in Chinese patients with epithelial ovarian cancer: a prospective cohort study
Source: J Ovarian Res. 2019 Aug 31;12:80. doi: 10.1186/s13048-019-0560-y (PMC6717355; doi:10.1186/s13048-019-0560-y)
Supplement: Supplementary file 1 — Table S1. Cases with deleterious germline and somatic mutations. (DOCX 22 kb) [file 13048_2019_560_MOESM1_ESM.docx]

Additional file 1: Table S1. Cases with deleterious germline and somatic mutations.

| Stage | Grade | Histology | Tumor type | Age at diagnosis | Germline mutation(s) | Somatic mutation(s)^a^ |
| --- | --- | --- | --- | --- | --- | --- |
| IIIC | 3 | Serous | Primary | 62 | *STK11* | *TP53* |
| IVB | 3 | Serous | Recurrent | 58 | *RAD51C* | *TP53* |
| IIIC | 3 | Serous | Recurrent | 47 | *PALB2* | *TP53* |
| IIIC | 3 | Serous | Primary | 64 | *PALB2* |  |
| IIIB | 1 | Serous | Recurrent | 55 | *CHEK2* | *ATM* |
| IIIC | 3 | Serous | Primary | 62 | *BRCA2* | *TP53* |
| IIC | 3 | Serous | Primary | 56 | *BRCA1*, *BRCA2* | *TP53* |
| IIIC | 3 | Serous | Primary | 65 | *BRCA1* | *TP53*, *PTEN* |
| NA | 3 | Serous | Primary | 66 | *BRCA1* | *TP53* |
| IVB | 3 | Serous | Primary | 47 | *BRCA1* | *TP53* |
| IV | 3 | Serous | Primary | 63 | *BRCA1* | *TP53* |
| IV | 3 | Serous | Primary | 45 | *BRCA1* | *TP53* |
| IB | 3 | Serous | Recurrent | 54 | *BRCA1* | *TP53* |
| IV | 3 | Serous | Primary | 45 | *BRCA1* |  |
| IV | 3 | Serous | Primary | 42 | *BRCA1* |  |
| IIIC | 3 | Serous | Primary | 63 | *BRCA1* |  |
| IIIC | 3 | Serous | Primary | 45 | *BRCA1* |  |
| IIIC | 3 | Serous | Recurrent | 53 | *BRCA1* |  |
| IIIC | 3 | Serous | Primary | 55 |  | *TP53*, *BRCA1* |
| III | 3 | Serous | Primary | 52 |  | *TP53*, *BRCA1* |
| NA | 3 | Serous | Recurrent | 57 |  | *TP53* |
| NA | 1 | Mixed | Primary | 63 |  | *TP53* |
| NA | 3 | Serous | Recurrent | 52 |  | *TP53* |
| IVA | 3 | Serous | Primary | 70 |  | *TP53* |
| IV | 3 | Serous | Primary | 46 |  | *TP53* |
| IIIC | 3 | Serous | Primary | 59 |  | *TP53* |
| IIIC | 3 | Serous | Primary | 57 |  | *TP53* |
| IIIC | 3 | Serous | Primary | 39 |  | *TP53* |
| IIIC | 3 | Serous | Primary | 41 |  | *TP53* |
| IIIC | 3 | Serous | Primary | 60 |  | *TP53* |
| IIIC | 3 | Serous | Primary | 48 |  | *TP53* |
| IIIC | 3 | Serous | Primary | 77 |  | *TP53* |
| IIIC | 3 | Serous | Primary | 82 |  | *TP53* |
| IIIC | 3 | Serous | Primary | 52 |  | *TP53* |
| IIIC | 3 | Serous | Primary | 47 |  | *TP53* |
| IIIC | 3 | Serous | Primary | 40 |  | *TP53* |
| IIIB | 3 | Endometrioid | Recurrent | 65 |  | *TP53* |
| III | 3 | Serous | Recurrent | 51 |  | *TP53* |
| III | 3 | Serous | Primary | 68 |  | *TP53* |
| IIB | 3 | Serous | Primary | 60 |  | *TP53* |
| IC | 3 | Serous | Recurrent | 42 |  | *TP53* |
| IC | NA | Malignant Brenner | Recurrent | 34 |  | *TP53* |
| IIIC | NA | Clear cell | Recurrent | 48 |  | *PTEN* |
| IC | NA | Endometrioid | Recurrent | 49 |  | *PTEN* |
| IIIC | 3 | Serous | Primary | 65 |  | *BRCA2* |
| IIIC | 3 | Serous | Primary | 47 |  | *BRCA1* |
| NA | NA | Mucinous | Primary | 82 |  | *ATM* |
| IC | NA | Clear cell | Recurrent | 41 |  | *ATM* |
| NA | 3 | Squamous cell | Primary | 54 |  |  |
| IV | 3 | Serous | Primary | 52 |  |  |
| IIIC | 3 | Serous | Primary | 62 |  |  |
| IIIC | 3 | Unclassified adenocarcinoma | Primary | 43 |  |  |
| IIIC | 3 | Serous | Primary | 64 |  |  |
| IIIC | 3 | Serous | Primary | 58 |  |  |
| IIIC | 3 | Serous | Primary | 67 |  |  |
| IIIC | 3 | Serous | Primary | 56 |  |  |
| IIIC | 3 | Serous | Primary | 59 |  |  |
| IIIB | 3 | Serous | Primary | 51 |  |  |
| IIB | NA | Clear cell | Primary | 69 |  |  |
| IIB | NA | Clear cell | Primary | 48 |  |  |
| IIB | 3 | Endometrioid | Primary | 66 |  |  |
| IC | NA | Serous | Primary | 56 |  |  |

NA= not available/unknown. Dx= diagnosis.

^a^ Accession numbers are as follows: ATM, NM_000051; PTEN, NM_000314; TP53, NM_000546; BRCA1, NM_007294; BRCA2, NM_000059.
