# Supplementary material for: Germline and somatic mutations of multi-gene panel in Chinese patients with epithelial ovarian cancer: a prospective cohort study
Source: J Ovarian Res. 2019 Aug 31;12:80. doi: 10.1186/s13048-019-0560-y (PMC6717355; doi:10.1186/s13048-019-0560-y)
Supplement: Supplementary file 2 — Table S2. Cases with deleterious mutations. (DOCX 28 kb) [file 13048_2019_560_MOESM2_ESM.docx]

Additional file 2: Table S2. Cases with deleterious mutations.

| **Stage** | **Grade** | **Histology** | **Somatic mutation(s)^a^** | **Amino acid change** | **Functional changes** | **Mutational type** | **Loss of heterozygosity** | **Germline mutation(s)** | **Amino acid change** | **Functional changes** | **Mutational type** |
| --- | --- | --- | --- | --- | --- | --- | --- | --- | --- | --- | --- |
| IIC | 3 | Serous | *TP53* c.641A>G | p.His214Arg | Missense | Likely pathogenic |  | *BRCA1* c.1214C>G  *BRCA2* c.3883C>T | p.Ser405Ter  p.Gln1295Ter | Nonsense  Nonsense | Pathogenic  Likely pathogenic |
| IC | 3 | Serous | *TP53* c.844C>T | p.Arg282Trp | Missense | Pathogenic | Yes |  |  |  |  |
| IC | NA | Malignant Brenner | *TP53* c.527G>T | p.Cys176Phe | Missense | Likely pathogenic |  |  |  |  |  |
| IIIC | 3 | Serous | *TP53* c.488A>G | p.Tyr163Cys | Missense | Likely pathogenic |  | *PALB2* c.3121A>T | p.Lys1041Ter | Nonsense | Likely pathogenic |
| IIIC | 3 | Serous | *TP53* c.536A>G | p.His179Arg | Missense | Likely pathogenic |  |  |  |  |  |
| IVA | 3 | Serous | *TP53* c.151delG | p.Glu51AsnfsX72 | Frameshift | Pathogenic |  |  |  |  |  |
| IIIC | 3 | Serous | *TP53* c.1027G>T | p.Glu343Ter | Nonsense | Pathogenic |  |  |  |  |  |
| IIIC | 3 | Serous | *TP53* c.711G>A | p.Met237Ile | Missense | Likely pathogenic |  |  |  |  |  |
| IIIC | 3 | Serous | *BRCA2* c.3460delA | p.Thr1154ProfsX14 | Frameshift | Likely pathogenic |  |  |  |  |  |
| IVB | 3 | Serous | *TP53* c.536A>T | p.His179Leu | Missense | Likely pathogenic |  | *BRCA1* EX18_19 Del | - | Deletion | Pathogenic |
| IIIC | 3 | Serous | *TP53* c.817C>T | p.Arg273Cys | Missense | Likely pathogenic |  | *STK11* EX1_10 DEL | - | Deletion | Pathogenic |
| IIIC | 3 | Serous | *TP53* c.625_626delAG | p.Arg209LysfsX6 | Frameshift | Likely pathogenic |  |  |  |  |  |
| IIIC | 3 | Serous | *TP53* c.709delA | p.Met237Cysfs*10 | Frameshift | Likely pathogenic |  |  |  |  |  |
| NA | 3 | Serous | *TP53* c.782+2_c.782+6delTCAGG | - | Splice | Likely pathogenic |  | *BRCA1* c.5470_5477delATTGGGCA | p.Ile1824AspfsX3 | Frameshift | Pathogenic |
| IIIC | 3 | Serous | *TP53* c.577C>T | p.His193Tyr | Missense | Likely pathogenic |  |  |  |  |  |
| IIB | 3 | Serous | *TP53* c.659A>G | p.Tyr220Cys | Missense | Likely pathogenic | Yes |  |  |  |  |
| NA | 3 | Serous | *TP53* c.524G>A | p.Arg175His | Missense | Pathogenic |  |  |  |  |  |
| IV | 3 | Serous | *TP53* c.700T>C | p.Tyr234His | Missense | Pathogenic |  | *BRCA1* c.212+3A>G | - | Splice | Pathogenic |
| IVB | 3 | Serous | *TP53* c.659A>G | p.Tyr220Cys | Missense | Likely pathogenic | Yes | *RAD51C* c.635delG | p.Arg212ProfsX27 | Frameshift | Likely pathogenic |
| IC | NA | Endometrioid | *PTEN* c.302_305delinsACC/c.406delT/  c.635-1G>T | p.Ile101AsnfsX12、p.Cys136ValfsX11、- | Frameshift/Frameshift  Splice | Likely pathogenic |  |  |  |  |  |
| IV | 3 | Serous | *TP53* c.916C>T | p.Arg306Ter | Nonsense | Pathogenic |  | *BRCA1* c.4986+1G>C | - | Splice | Likely pathogenic |
| IB | 3 | Serous | *TP53* c.743G>A | p.Arg248Gln | Missense | Pathogenic |  | *BRCA1* c.5092G>T | p.Glu1698Ter | Nonsense | Pathogenic |
| IIIC | 3 | Serous | *BRCA1* c.3133_3134delAA | p.Asn1045* | Nonsense | Likely pathogenic |  |  |  |  |  |
| IIIC | 3 | Serous | *TP53* c.659A>G | p.Tyr220Cys | Missense | Likely pathogenic |  |  |  |  |  |
| IIIC | 3 | Serous | *TP53* c.584T>A | p.Ile195Asn | Missense | Likely pathogenic |  | *BRCA2* c.5578A>T | p.Lys1860Ter | Nonsense | Pathogenic |
| IIIC | 3 | Serous | *TP53* c.661G>T | p.Glu221Ter | Nonsense | Likely pathogenic | Yes |  |  |  |  |
| III | 3 | Serous | *TP53* c.600delT  *BRCA1* c.2760_2763delACAG | p.Leu201Cysfs*46  p.Thr922Leufs*77 | Frameshift  Frameshift | Likely pathogenic  Pathogenic |  |  |  |  |  |
| NA | 1 | Mixed | *TP53* c.532C>G | p.His178Asp | Missense | Likely pathogenic | Yes |  |  |  |  |
| III | 3 | Serous | *TP53* c.839G>C | p.Arg280Thr | Missense | Likely pathogenic |  |  |  |  |  |
| IIIC | 3 | Serous | *TP53* c.994-2A>G | - | Splice | Pathogenic |  |  |  |  |  |
| IIIC | 3 | Serous | *TP53* c.713G>T  *BRCA1* c.3380_3402delATCTGATTTCAGATAACTTAGAA | p.Cys238Phe  p.Tyr1127SerfsX6 | Missense  Frameshift | Likely pathogenic |  |  |  |  |  |
| IIIB | 1 | Serous | *ATM* c.2458_c.2466+8delAAAAGTTTAGTAAGTAT | - | Span | Likely pathogenic |  | *CHEK2* c.1111C>T | p.His371Tyr | Missense | Likely pathogenic |
| NA | NA | Mucinous | *ATM* c.748C>T | p.Arg250Ter | Nonsense | Pathogenic |  |  |  |  |  |
| IIIC | 3 | Serous | *TP53* c.673-2A>T | - | Splice | Likely pathogenic |  |  |  |  |  |
| III | 3 | Serous | *TP53* c.775G>T | p.Asp259Tyr | Missense | Likely pathogenic | Yes |  |  |  |  |
| IV | 3 | Serous | *TP53* c.96+1G>T | - | Splice | Likely pathogenic |  |  |  |  |  |
| IIIC | 3 | Serous | *TP53* c.213_215delCCCinsGC  *PTEN* c.616_632delTTCAGTGGCGGAACTTG | p.Val73Trpfs  p.Phe206GlnfsX31 | Frameshift  Frameshift | Pathogenic  Likely pathogenic |  | *BRCA1* c.4801A>T | p.Lys1601Ter | Nonsense | Pathogenic |
| IIIB | 3 | Endometrioid | Pathogenic | p.Pro152Argfs*18 | Frameshift | Pathogenic |  |  |  |  |  |
| IIIC | NA | Clear cell | *PTEN* c.797_c.801+4delAAAAGGTTT | - | Span | Likely pathogenic |  |  |  |  |  |
| IC | NA | Clear cell | *ATM* c.8343_8344delTA | p.Asn2782Glnfs*2 | Frameshift | Likely pathogenic |  |  |  |  |  |
| IIIC | 3 | Serous | *TP53* c.742C>T | p.Arg248Trp | Missense | Pathogenic |  |  |  |  |  |
| NA | 3 | Serous | *TP53* c.499C>T | p.Gln167Ter | Nonsense | Likely pathogenic |  |  |  |  |  |
| IIIC | 3 | Serous |  |  |  |  |  | *PALB2* c.246_247insA | p.His83Thrfs*7 | Frameshift | Likely pathogenic |
| IV | 3 | Serous |  |  |  |  |  | *BRCA1* c.3756_3759 delGTCT | p.Ser1253Argfs*10 | Frameshift | Pathogenic |
| IV | 3 | Serous |  |  |  |  |  | *BRCA1* c.520C>T | p.Gln174Ter | Nonsense | Pathogenic |
| ⅢC | 3 | Serous |  |  |  |  |  | *BRCA1* c.2751delC | p.Lys918Serfs*82 | Frameshift | Likely pathogenic |
| IIIC | 3 | Serous |  |  |  |  |  | *BRCA1* c.1660G>T | p.Glu554Ter | Nonsense | Pathogenic |
| IIIC | 3 | Serous |  |  |  |  |  | *BRCA1* c.5095C>T | p.Arg1699Trp | Missense | Likely pathogenic |
