# Supplementary material for: Germline and somatic mutations of multi-gene panel in Chinese patients with epithelial ovarian cancer: a prospective cohort study
Source: J Ovarian Res. 2019 Aug 31;12:80. doi: 10.1186/s13048-019-0560-y (PMC6717355; doi:10.1186/s13048-019-0560-y)
Supplement: Supplementary file 3 — Table S3. Cases with familial and personal histories of HBOC-related tumors. (DOCX 16 kb) [file 13048_2019_560_MOESM3_ESM.docx]

Additional file 3: Table S3. Cases with familial and personal history of HBOC-related tumors.

| **Stage** | **Grade** | **Histology** | **Family history** | **Somatic mutation(s)** | **Germline mutation(s)** |
| --- | --- | --- | --- | --- | --- |
| IIIC | 3 | Serous | Personal |  | *BRCA1* |
| IIIC | 3 | Serous | Personal+Familial | *TP53* | *BRCA1* |
| IC | 3 | Serous | Personal | *TP53* |  |
| IC | 3 | Serous | Personal | *TP53* |  |
| IIIC | 3 | Serous | Personal | *TP53* |  |
| NA | 3 | Serous | Familial |  | *BRCA1* |
| NA | 3 | Endometrioid | Familial |  |  |
| IB | 3 | Serous | Familial |  | *BRCA1* |
| III | 3 | Serous | Familial |  | *BRCA1* |
| IIC | 3 | Serous | Familial | *TP53* | *BRCA1* |
| IIIC | 3 | Serous | Familial | *TP53* | *BRCA1*+*BRCA2* |
| IIIC | 3 | Serous | Familial | *TP53* | *BRCA1* |
| III | 3 | Serous | Familial | *TP53* | *BRCA1* |
| IVB | 3 | Serous | Familial | *TP53* |  |
